# A General Strategy to Fabricate Carbon‐Coated 3D Porous Interconnected Metal Sulfides: Case Study of SnS/C Nanocomposite for High‐Performance Lithium and Sodium Ion Batteries

**DOI:** 10.1002/advs.201500200

**Published:** 2015-09-02

**Authors:** Changbao Zhu, Peter Kopold, Weihan Li, Peter A. van Aken, Joachim Maier, Yan Yu

**Affiliations:** ^1^ Max Planck Institute for Solid State ResearchHeisenbergstr. 1Stuttgart70569Germany; ^2^ CAS Key Laboratory of Materials for Energy ConversionDepartment of Materials Science and EngineeringUniversity of Science and Technology of ChinaAnhuiHefei230026P. R. China

**Keywords:** 3D porous interconnected composite, electrostatic spray deposition (ESD), lithium storage, metal sulfides, SnS, sodium storage

## Abstract

Transition metal sulfides have a great potential for energy storage due to the pronouncedly higher capacity (owing to conversion to metal or even alloy) than traditional insertion electrode materials. However, the poor cycling stability still limits the development and application in lithium and sodium ion batteries. Here, taking SnS as a model material, a novel general strategy is proposed to fabricate a 3D porous interconnected metal sulfide/carbon nanocomposite by the electrostatic spray deposition technique without adding any expensive carbonaceous materials such as graphene or carbon nanotube. In this way, small nanorods of SnS are generated with sizes of ≈10–20 nm embedded in amorphous carbon and self‐assembled into a 3D porous interconnected nanocomposite. The SnS:C is directly deposited on the Ti foil as a current collector and neither conductive additives nor binder are needed for battery assembly. Such electrodes exhibit a high reversible capacity, high rate capability, and long cycling stability for both lithium and sodium storage.

## 1 Introduction

This is an open access article under the terms of the Creative Commons Attribution License, which permits use, distribution and reproduction in any medium, provided the original work is properly cited.

In order to meet the requirements for grid‐scale energy storage as well as for electric vehicles, the energy densities of lithium ion batteries (LIBs) need to be further improved, over the energy density that conventional LIBs based on LiMO_2_ (M = transition metal) cathodes and graphite anodes can reach.^1^ In this context, transition metal compounds, such as oxides and sulfides, have attracted great attention.^2^, ^3^ Among them, metal sulfides based on conversion to metal or even alloy can offer higher capacities than traditional intercalation compounds through transferring more than one electron per metal ion, but suffer from less volumetric expansion on lithiation compared with Si or metal oxides.^4^

Recently, sodium ion batteries (SIBs) are considered to be one of the realistic alternatives to LIBs, because of the abundance of sodium sources, lower environmental impact, and the more favorable cost issues.^5^ However, due to the inherent larger radius of Na^+^ (0.102 nm) compared with Li^+^ (0.076 nm), it results in more severe volume expansion, which brings about unsolved challenges for SIBs.

Transition metal sulfides MS*_x_* (M = Fe, Co, Ni, Mn, Cu, etc.) have been investigated both as cathode and anode materials for high‐energy LIBs since 1970s.^4^, ^6^ Especially, layered transition metal sulfides of MS*_x_* (M = Mo, W, V, Sn, Zr) have attracted great attention due to their structure peculiarities and redox variabilities, resulting in much better cycling stability than other metal sulfides exhibit.^4^, ^7^, ^8^, ^9^, ^10^, ^11^ Furthermore, transition metal sulfides have been investigated for sodium storage as well.^12^, ^13^, ^14^, ^15^, ^16^, ^17^, ^18^, ^19^ Their superior electrochemical performance makes them highly promising candidates for sodium anode materials.

Nonetheless, there are also some disadvantages for metal sulfides in the context of lithium and sodium storage. The storage mechanism of metal sulfides is based on the conversion reaction to form Li_2_S (Na_2_S) and the transition metal or, if the transition metal can take up Li or Na (such as SnS_2_ and SnS), to a combination of conversion and alloying reaction. Owing to this complexity, there are several inherent issues. First of all, one faces severe kinetic problems in terms of reactivity, mass transport, and nucleation due to the decomposition into multiphases.^13^ Next, for both conversion reaction and alloying process, large volume changes occur during charge–discharge cycles, leading to serious agglomeration of electrode particles, if not pulverization and electric disconnection from current collectors, which are major reasons for the poor cycling stability.^4^ Furthermore, the electronic conductivities of metal sulfides are low,^20^ which is connected with a poor rate capability. Different concepts of nanostructure design have been applied to overcome these problems. One way is to decrease the size of metal sulfide and optimize the morphology in order to reduce the transport length of ions.^7^, ^21^, ^22^, ^23^, ^24^, ^25^, ^26^, ^27^ The other method is to prepare carbon‐coated metal sulfides or metal sulfides/carbon composites so as to increase the electronic conductivity and buffer the volume change during cycling.^28^, ^29^, ^30^, ^31^, ^32^, ^33^, ^34^, ^35^ Among the carbonaceous materials, graphene and carbon nanotubes (CNT) are commonly used, which are found to be very efficient to increase the performance.^36^, ^37^, ^38^ However, the cost of graphene and CNTs and the complicated processing steps limit the feasibility of such method in real large‐scale application. Moreover, most of such metal sulfides/graphene (or CNTs) composites are prepared by a hydrothermal approach. They must be complemented by a standard but tedious electrode preparation, viz., casting slurries consisting of active materials, binders, and conductive additives on current collectors. All in all, a viable method is to fabricate small mechanically isolated but electrochemically well‐connected metal sulfides particles in a conductive matrix without using expensive graphene or CNT.

Electrostatic spray deposition (ESD) is a versatile approach to engineer electrode materials with various morphologies, such as dense, porous, and sponge‐like ones.^39^ In view of the application as battery materials, a 3D porous composite is quite promising. The other advantage of this technique is that electrode materials can be directly deposited on the current collectors without any binder or conductive additives, greatly simplifying the battery fabrication process. A large number of oxidic electrode materials have been prepared by the ESD technique, i.e., TiO_2_, Fe_2_O_3_, CoO, SnO_2_, etc.,^40^, ^41^, ^42^, ^43^ yet application to metal sulfides is quite rare.^44^ Recently, we have successfully prepared layered metal sulfides (such as MoS_2_ and WS_2_) and metal sulfide–graphene–CNT nanocomposites for lithium and sodium storage.^44^, ^45^ For this purpose, (NH_4_)_2_MoS_4_ and (NH_4_)_2_WS_4_ precursors have been chosen, which easily decompose to MoS_2_ or WS_2_ according to: ${\left({\mathrm{NH}}_{4}\right)}_{2}{\mathrm{MoS}}_{4}+H_{2}\to {\mathrm{MoS}}_{2}+H_{2}S+{\mathrm{NH}}_{3}$ or ${\left({\mathrm{NH}}_{4}\right)}_{2}{\mathrm{WS}}_{4}+H_{2}\to {\mathrm{WS}}_{2}+H_{2}S+{\mathrm{NH}}_{3}$. However, the success relies on the choice of the precursor, which limits the applicability to other metal sulfides. Hence, it is desirable to design a generalizable strategy for fabricating 3D porous metal sulfides by ESD for lithium and sodium storage.

Here, we take SnS as an example, to demonstrate and verify our general strategy to prepare carbon‐coated 3D porous interconnected metal sulfides. Small nanorods of SnS (≈10–20 nm) are embedded in amorphous carbon and self‐assemble into a 3D porous interconnected nanocomposite. The SnS/C nanocomposite (without using any expensive carbonaceous materials, such as graphene and CNTs) is directly deposited on the Ti foil as a current collector, and neither conductive additives nor binder are needed for battery assembling. Such an SnS electrode exhibits superior electrochemical performance for both lithium and sodium storage.

## 2 Results and Discussions

**Figure 1:**
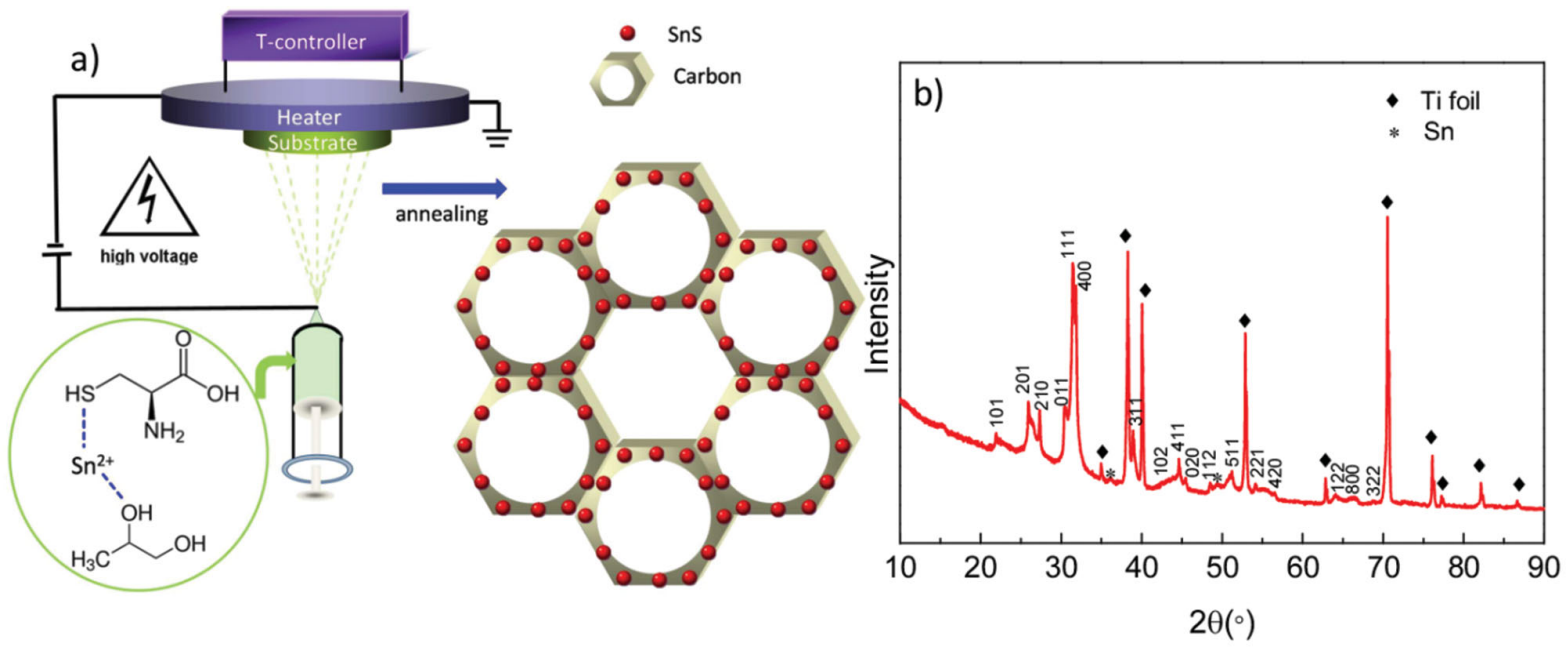
a) Schematic illustration of ESD technique to fabricate a carbon‐coated 3D porous interconnected SnS. b) XRD pattern of such SnS/C nanocomposite.

Our strategy to prepare 3D porous metal sulfides by ESD is to choose l‐cysteine together with a metal ion source (here SnCl_2_). **Figure** **1**a schematic illustrates how to design and construct carbon‐coated 3D porous SnS/C nanocomposites (abbreviated as SnS:C). A typical ESD setup includes mainly three parts: the precursor‐providing system, where a nozzle is connected to a syringe to provide the precursor solution at a constant flow rate; the heated substrate, whose temperature will be controlled by a thermo couple; and the high DC voltage supply.^39^ The l‐cysteine is not only the sulfur source for sulfide formation, but it also acts as a complexing agent for the Sn^2+^ ion in the 1,2‐propanediol solvent. The l‐cysteine molecule has different functional groups, including –NH_2_, –COOH, and –SH, exhibiting a strong tendency to coordinate inorganic cations, such as Sn^2+^.^46^, ^47^ During the ESD process, the precursor solution is atomized into aerosol (composed of the charged droplets) on applying a high voltage between the nozzle and substrate. The charged droplets are then attracted to and deposited on the heated substrate. The polyol oligomers formed by 1,2‐propanediol are evaporated at high temperature and give rise to a large number of voids in the droplets, finally leading to 3D porous interconnected structure.^48^ Meanwhile, the Sn^2+^–l‐cysteine complex decomposes to form SnS. After annealing in Ar, nanorods of crystalline SnS form and the extra l‐cysteine and remaining 1,2‐propanediol transform to amorphous carbon. Finally, a carbon‐coated 3D porous interconnected SnS is formed. The X‐ray diffraction (XRD) pattern of SnS:C is shown in Figure 1b. The most pronounced peaks are well indexed to the orthorhombic SnS phase (JPCDS no. 39‐0354), and only two very weak peaks around 36° and 49° (2*θ*) are identified to correspond to trace amount of Sn metal. The strong Ti signal stems from the Ti metal foil, used as a growth substrate and battery current collector due to its high chemical stability compared to the conventionally used current collectors.

**Figure 2:**
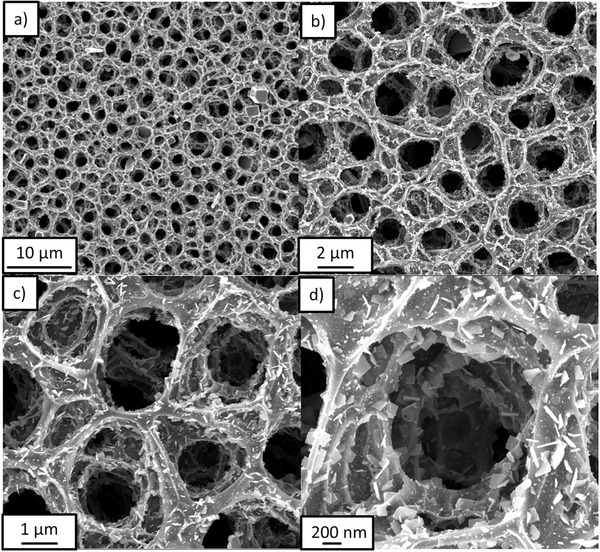
a–d) SEM images of carbon‐coated 3D porous interconnected SnS at different magnifications.

The morphology and microstructure of SnS/C nanocomposites were investigated via scanning electron microscopy (SEM). **Figure** **2**a–d displays SEM images for SnS:C at various magnifications. The lower magnifications (Figure 2a,b) clearly demonstrate that the whole deposited composite exhibits the 3D porous interconnected structure. The average pore size is ≈2 μm and the thickness of the pore wall is around hundred nm. The large pores are mainly due to solvent evaporation during the ESD process. At higher magnifications (Figure 2c,d), the detailed microstructure of SnS/C composite can be seen clearly. The walls of pores are not smooth but rather rough and hierarchical pores appear with sizes of hundred nm. Excess l‐cysteine and remaining solvent decompose into carbon, and produce even more pores during the annealing process. The nanoplates and nanoparticles are embedded or partially embedded in walls of such 3D porous interconnected composite. To investigate the chemical composition of SnS/C nanocomposite, energy‐dispersive X‐ray (EDX) analysis was applied (Figure S1, Supporting Information). It was found that the atomic ratios of Sn:S:C are 1:1.04:5.68, which is corresponding to the carbon content of 31 wt% in the SnS/C nanocomposite. During ESD process, without using l‐cysteine in precursor, SnO_2_/C is obtained instead of SnS/C; without using SnCl_2_ in precursor, only carbon is obtained. Both SnO_2_/C composite and carbon exhibit 3D porous interconnected structure as shown in Figures S2 and S3 (Supporting Information).

**Figure 3:**
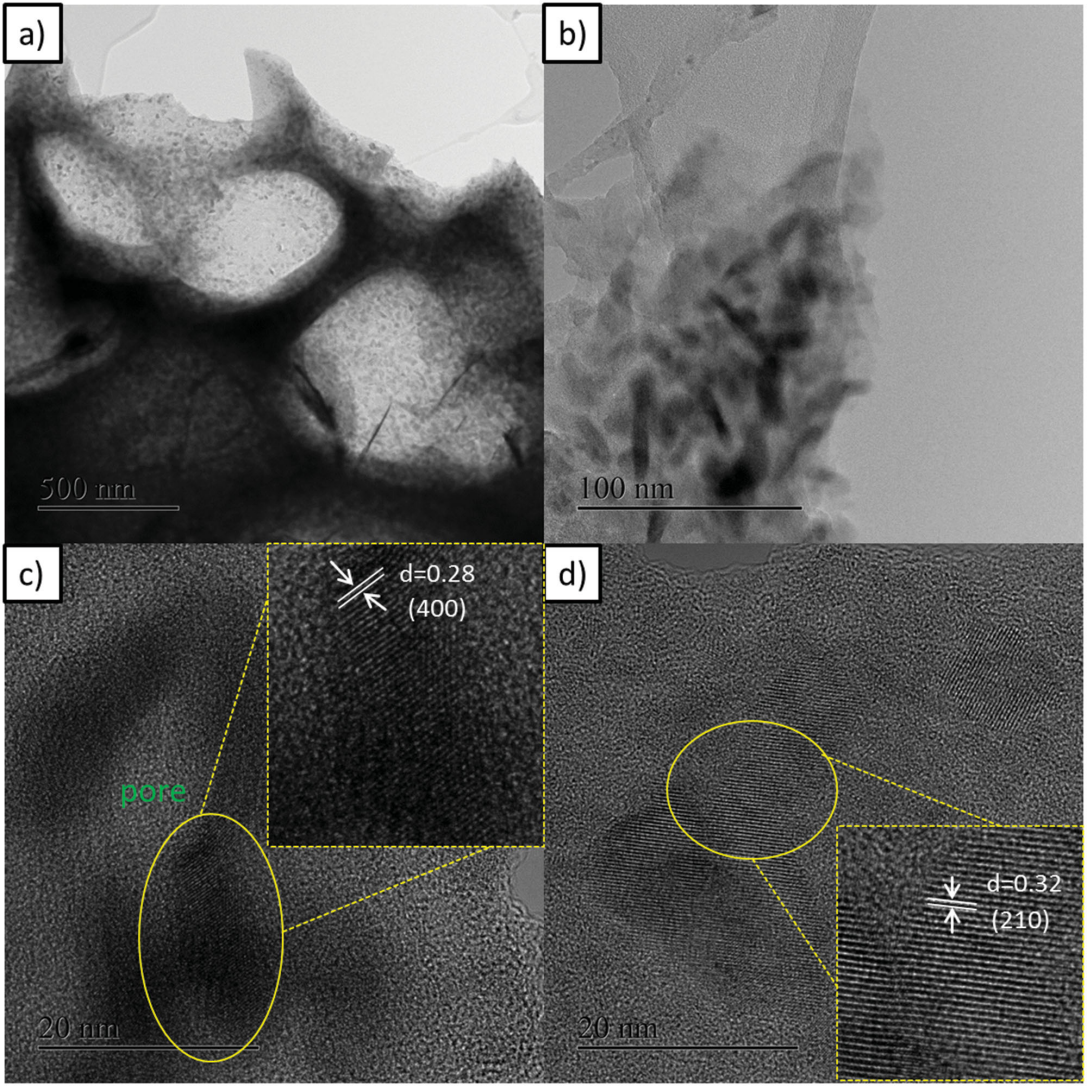
a,b) TEM‐BF images of 3D porous interconnected SnS/C nanocomposite at different magnifications. c,d) HRTEM images of 3D porous interconnected SnS/C nanocomposite.

In order to further investigate the morphology of the nanocomposite, transmission electron microscopy (TEM) and high‐resolution TEM (HRTEM) were applied. As shown in **Figure** **3**a, the 3D porous interconnected structure is clearly identified, which is consistent with SEM images. Furthermore, it displays that such 3D porous interconnected composite (as well as for nanoplates appearing on the surface, Figure S4, Supporting Information) is constructed by tiny SnS nanoparticles, which are uniformly embedded in the amorphous carbon. This unique structure will not only increase the electronic conductivity of the composite, but also effectively buffer the volume change during charge–discharge cycling. The shape of SnS is rod‐like, as shown in Figure 3b, with diameters of around 5–10 nm and lengths of ≈20 nm, and there are some pores between these nanorods, which can be found in Figure 3c, too. HRTEM images (Figure 3c,d) display clear lattice fringes with *d*‐spacings of ≈0.28 and ≈0.32 nm, respectively, which are attributed to the (400) and (210) lattice planes of the orthorhombic SnS phase, demonstrating a high degree of crystallinity. The presence of carbon and quality of carbon can be confirmed and evaluated by Raman spectra (Figure S5, Supporting Information). The D‐band at around 1375 cm^−1^ is assigned to structural disorder and imperfection, and the G‐band localized at ≈1580 cm^−1^ is attributed to ordered graphite phase.^49^ The respective Raman intensities of *I*_D_ and *I*_G_ are proportional to the numbers of scattering disordered and ordered carbon atoms, respectively. As a result, the ratio of *I*_D_/*I*_G_ is usually used to determine the crystallinity degree of carbon.^40^ The *I*_D_/*I*_G_ ratio for 3D porous SnS/C nanocomposite is 0.88, indicates that the majority of the carbon is of sp^2^‐type carbon, and hence providing high electronic conductivity. In order to investigate the chemical states of SnS/C nanocomposite, X‐ray photoelectron spectroscopy (XPS) was performed. C 1s signal at 284.8 eV is standardized as reference peak for the binding energies obtained in the XPS analysis. The Sn 3d peaks at 486.8 and 495.2 eV correspond to the binding energy of Sn 3d_5/2_ and Sn 3d_3/2_, and the peak of 161.7 eV is attributed to S 2p_3/2_ of SnS (Figure S6, Supporting Information). These results are in good agreement with reported values for SnS in the literatures.^50^, ^51^

**Figure 4:**
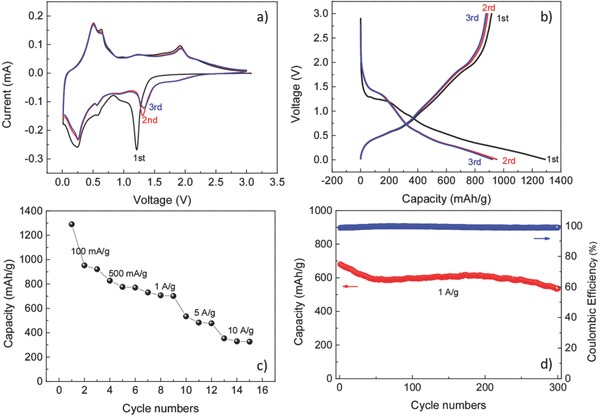
Electrochemical performance of carbon‐coated 3D porous interconnected SnS for lithium storage. a) Cyclic voltammograms at scan rate of 0.1 mV s^−1^. b) Charge and discharge voltage profiles for the first three cycles at current density of 100 mA g^−1^. c) Rate performance. d) Cycling performance and Coulombic efficiency at current density of 1 A g^−1^ cycling.

The electrochemical performance of SnS/C nanocomposite in terms of lithium storage is investigated by directly growing carbon‐coated SnS on the Ti current collectors without any conductive additives and binder. (In this work, if not specially mentioned, the electrochemical performance is obtained from the SnS:C composite with the ratio of 69: 31 by weight.) In order to study the electrochemical process of SnS:C, cyclic voltammetry (CV) is performed at first. **Figure** **4**a shows the CV curves for the first three cycles in terms of lithium storage in the voltage range of 0.01–3 V at a scan rate of 0.1 mV s^−1^, revealing similar electrochemical reactions features as discussed in the literature.^33^, ^52^ The strong peak at ≈1.2 V in the first cathodic cycle, is attributed to the conversion reaction of SnS to form metallic Sn and Li_2_S. The two peaks around ≈0.57 and ≈0.24 V correspond to the subsequent alloying reaction of Sn with Li. In the first anodic sweep, the peaks located at ≈0.50 and ≈0.64 V are attributed to the dealloying process of Li*_x_*Sn. The peak around 1.9 V can be assigned to the back‐conversion of Sn and Li_2_S into SnS. During the following cycles, the redox peaks appear at similar positions with similar profiles, indicating highly reversible storage reactions. Figure 4b shows the charge–discharge profiles of SnS:C in terms of lithium storage for the first three cycles at a current density of 100 mAh g^−1^. The inconspicuous voltage plateaus in the galvanostatic charge–discharge profiles are consistent with the electrochemical reactions recognized in the CV curves. In the first cycle, the discharge and charge capacity is 1290 and 916 mAh g^−1^, respectively. The irreversible portion can be attributed to the solid electrolyte interface (SEI) formation. Note that all the capacity values in this work are based on the total mass of the SnS/C composite. Since the theoretical capacities for SnS (1137 mAh g^−1^) and Sn (990 mAh g^−1^) are similar, effect of trace Sn on the specific capacity of the composite is negligible. As Figure 4c shows the rate capability is excellent. The specific discharge capacities are 953, 776, and 701 mAh g^−1^ at current densities of 100, 500, and 1000 mA g^−1^, respectively. Even at very high current densities, such as 5 and 10 A g^−1^, the specific capacities are still as high as 484 and 329 mA g^−1^. Furthermore, carbon‐coated 3D porous SnS also exhibits an excellent cycling stability. In the literature, the cycling performance of SnS in terms of lithium storage is usually investigated for less than 100 cycles, which is not very meaningful as far as long cycling behavior is concerned. In this work, the specific capacity of SnS:C is around 607 mAh g^−1^ after 200 cycles at current density of 1 A g^−1^, corresponding to ≈90% initial capacity. Even after 300 cycles, the capacity is still as high as 535 mAh g^−1^, with ≈80% capacity retention and high Coulombic efficiency (Figure 4d). In principle, increase porosity of electrode is able to improve the gravimetric capacity but decrease volumetric capacity. For our 3D porous composite, the volumetric specific capacity is estimated to be 620 mAh cm^−3^, given the loading mass of ≈0.6–0.8 mg cm^−2^ and average electrode thickness of ≈10 μm. This good electrochemical performance demonstrates the feasibility of ESD‐prepared carbon‐coated 3D porous interconnected SnS as high rate and long cycling lithium battery anode.

**Figure 5:**
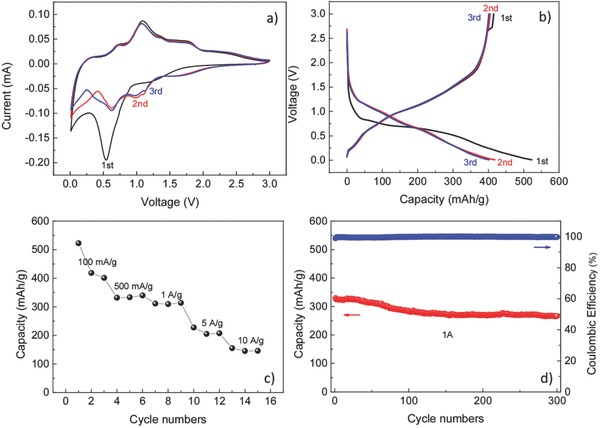
Electrochemical performance of carbon‐coated 3D porous interconnected SnS for sodium storage. a) Cyclic voltammograms at scan rate of 0.1 mV s^−1^. b) Charge and discharge voltage profiles for the first three cycles at current density of 100 mA g^−1^. c) Rate performance. d) Cycling performance and Coulombic efficiency at current density of 1 A g^−1^ cycling.

In addition, the sodium storage behavior of this electrode was investigated as well. CV was carried out first to clarify the corresponding electrochemical reactions in terms of sodium storage. **Figure** **5**a shows the CV curves for the first three cycles in the voltage range of 0.01–3 V at a scan rate of 0.1 mV s^−1^. During the first reduction process, a strong peak at around 0.54 V is attributed to SEI formation. After the first cycle, the reduction peaks localized at ≈0.01 and ≈0.63 V correspond to the alloying process, while the oxidation peaks at around 0.31 and 0.71 V can be attributed to the dealloying. The reduction peak at ≈0.98 V can be assigned to the conversion reaction to form Na_2_S and Sn metal, and the oxidation peak localized at around 1.06 V corresponds to the back‐conversion of Sn and Na_2_S into the SnS phase. The galvanostatic charge and discharge curves for the first three cycles for the sodium storage at current density of 100 mAh g^−1^ are consistent with the CV curves (Figure 5b). The initial discharge and charge capacities are 523 and 415 mAh g^−1^, respectively. The Coulombic efficiency of the first cycle is around 79%, which is much higher than that of oxides for the sodium storage.^53^ The irreversible capacity is due to SEI formation, which is usually observed for anode materials especially for the nanostructure anodes. The Coulombic efficiency increases to 97% during the second cycle, and further increases to 100% for the third cycle. The superior rate performance of SnS:C in terms of sodium storage is displayed in Figure 5c. The discharge capacities are 419, 334, 310, 205, and 145 mAh g^−1^, respectively, when current densities of 100, 500, 1000, 5000, and 10 000 mA g^−1^ are applied. As mentioned in the Introduction, it is usually very difficult to achieve long cycling sulfide anodes especially for sodium storage, owing to the large volume changes during cycling. In the literature, most reports for SnS only show less than 100 charge–discharge cycles. In this work, we demonstrate that our carbon‐coated 3D porous interconnected SnS exhibits excellent cycling stability (Figure 5d) for at least 300 cycles. The specific discharge capacities are 282, 270, and 266 mAh g^−1^, respectively, at current density of 1 A g^−1^ after 100, 200, and 300 cycles. After 300 cycles, the discharge capacity still maintains more than 80% capacity retention with almost ≈100% Coulombic efficiency.

Different SnS contents in the SnS/C nanocomposites are investigated. The electrochemical performances of pure carbon (0 wt% SnS) and the composite with 82 wt% SnS in terms of lithium and sodium storage are shown in Figures S7 and S8 (Supporting Information). When the SnS content increases from 69 to 82 wt%, the reversible capacities for both lithium and sodium storage increase as well (Figure S9, Supporting Information).

The excellent electrochemical performance of carbon‐coated 3D porous interconnected SnS prepared by ESD for both lithium and sodium storage can be attributed to its unique structure and morphology. (1) The tiny dimensions of the SnS particles (≈10–20 nm) are beneficial for the energy storage by improving the transport of both electrons and ions. (2) Uniformly coated carbon pronouncedly increases the electronic conductivity of the SnS:C. (3) The 3D porous interconnected structure not only effectively buffers the volume change during charge–discharge cycling, but also provides a large number of channels to give accesses to the electrolyte, which is facile for ion transport. (Note that these merits are even more important when sodium storage is concerned due to the inherent larger Na^+^ ion.) Moreover, there are several additional advantages of this material: these composites can be directly grown on the current collectors without any conductive carbon or binder, which greatly simplifies the battery fabrication process and appears to be particularly appropriate for thin film batteries. The SnS/C composite is prepared without any expensive and complex carbonaceous materials, such as graphene or CNT. Furthermore, the strategy is rather general, and can be easily applied to prepare other carbon‐coated metal sulfides, such as ZnS/C composite (Figure S10, Supporting Information).

## 3 Conclusions

In conclusion, we propose a general and novel strategy to fabricate a 3D porous interconnected metal sulfide/carbon nanocomposite by ESD technique without adding any expensive carbonaceous materials (i.e., graphene or CNT). Taken SnS as a model material, the small nanorods of SnS with sizes of ≈10–20 nm are embedded in amorphous carbon and self‐assemble into a 3D porous interconnected nanocomposite, which is directly deposited on a Ti foil that acts as current collector. Neither conductive additives nor binders are needed for battery assembly. Such SnS/C composites provide a high reversible capacity, rate capability, and long cycling stability for both lithium and sodium storage. As this simple but versatile preparation procedure can be easily applied to other metal sulfides, its potential for generating high‐performance electrodes for Li or Na storage is obvious.

## 4 Experimental Section

*Synthesis*: SnCl_2_ (0.005 m) and l‐cysteine (0.05 m) were dissolved to solvent of 1,2‐propanediol by stirring for 24 h. The resultant precursor solution was poured into a syringe connected to a metal nozzle of 1.6 mm diameter. The flow rate was ≈30 μL min^−1^. The distance between the substrate and metal nozzle was around 4 cm. Imporous Ti foil was chosen as a substrate and a current collector. Note that the porosity of current collector will have a significant effect on the volumetric specific capacities of the electrode. Before the deposition, the substrate was heated to 238 °C. A high voltage of 8 kV is applied for ESD. The as‐prepared SnS/C nanocomposites were annealed in a tube furnace at 550 °C for 2 h under Ar.

*Structural and Electrochemical Characterization*: XRD measurements were carried out with a Philips PW 3020 diffractometer using Cu Kα radiation. SEM was carried out using a JEOL 6300F field‐emission scanning electron microscopy (JEOL, Tokyo, Japan) operated at 15 keV. TEM and HRTEM were performed by using a JEOL 4000FX transmission electron microscope (JEOL, Tokyo, Japan) operated at 400 kV. XPS were performed on AXIS ULTRA spectrometer from Kratos Analytical Ltd.

*Electrochemical Test*: SnS/C nanocomposites grown on the Ti current collector used as working electrode were directly tested without binder or any conductive additives in a Swagelok‐type electrochemical test cell, which were assembled in an argon‐filled glove box (O_2_ ≤ 0.1 ppm, H_2_O ≤ 3 ppm). Sodium metal (lithium metal, for lithium battery) was used as the counter/reference electrode, and 1 m solution of NaClO_4_ in the propylene carbonate (PC) with 5% fluoroethylene carbonate (FEC) as the electrolyte (1 m solution of LiPF_6_ in a 1:1 vol/vol mixture of ethylene carbonate and diethyl carbonate as the electrolyte for lithium batteries). Glass fiber (Whatman) was used as separator. The batteries were discharged and charged galvanostatically in the voltage range between 0.01 and 3 V on an Arbin MSTAT battery tester at room temperature. Cyclic voltammetry was performed by Voltalab 80 electrochemical workstation at scan rate of 0.1 mV s^−1^.

## Supporting information

As a service to our authors and readers, this journal provides supporting information supplied by the authors. Such materials are peer reviewed and may be re‐organized for online delivery, but are not copy‐edited or typeset. Technical support issues arising from supporting information (other than missing files) should be addressed to the authors.

Supplementary
