# Supplementary material for: A General Strategy to Fabricate Carbon‐Coated 3D Porous Interconnected Metal Sulfides: Case Study of SnS/C Nanocomposite for High‐Performance Lithium and Sodium Ion Batteries
Source: Adv Sci (Weinh). 2015 Sep 2;2(12):1500200. doi: 10.1002/advs.201500200 (PMC5049589; doi:10.1002/advs.201500200)
Supplement: Supplementary file 1 — Supplementary [file ADVS-2-0r-s001.pdf]

Copyright WILEY-VCH Verlag GmbH & Co. KGaA, 69469 Weinheim, Germany, 2015.

ADVANCED SCIENCE
Open Access

Supporting Information

for *Adv. Sci.*, DOI: 10.1002/advs. 201500200

A General Strategy to Fabricate Carbon-Coated 3D Porous Interconnected Metal Sulfi des: Case Study of SnS/C Nanocomposite for High-Performance Lithium and Sodium Ion Batteries

*Changbao Zhu, Peter Kopold, Weihan Li, Peter A. van Aken, Joachim Maier, and Yan Yu**

Copyright WILEY-VCH Verlag GmbH & Co. KGaA, 69469 Weinheim, Germany, 2013.

Supporting information

A General Strategy to Fabricate Carbon Coated 3D Porous Interconnected Metal Sulfides: Case Study of SnS/C nanocomposite for High Performance Lithium and Sodium Ion Batteries

Changbao Zhu, Peter Kopold, Weihan Li, Peter A. van Aken, Joachim Maier and Yan Yu[*]

Prof. Yan Yu, Weihan Li
CAS Key Laboratory of Materials for Energy Conversion, Department of Materials Science and Engineering, University of Science and Technology of China, Hefei 230026, Anhui, P. R. China.
E-mail: yanyumse@ustc.edu.cn

Dr. Changbao Zhu, Mr. Peter Kopold, Prof. Peter A. van Aken, Prof. Yan Yu and Prof. Joachim Maier
Max Planck Institute for Solid State Research, Heisenbergstr. 1, Stuttgart, 70569, Germany

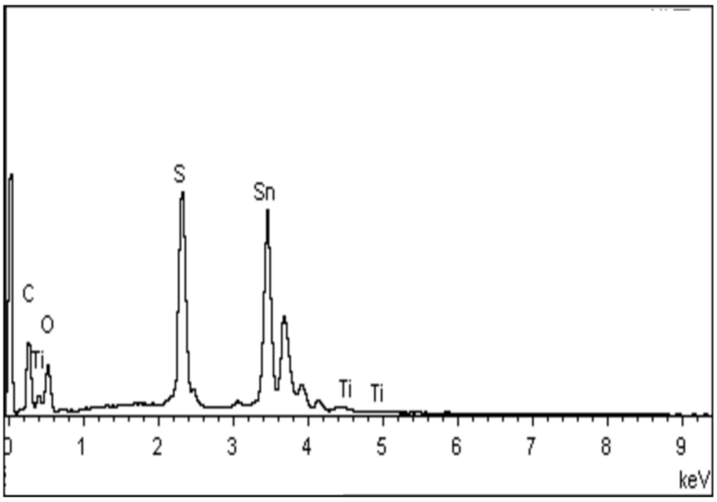

Figure S1. EDX spectra of carbon coated 3D porous interconnected SnS.

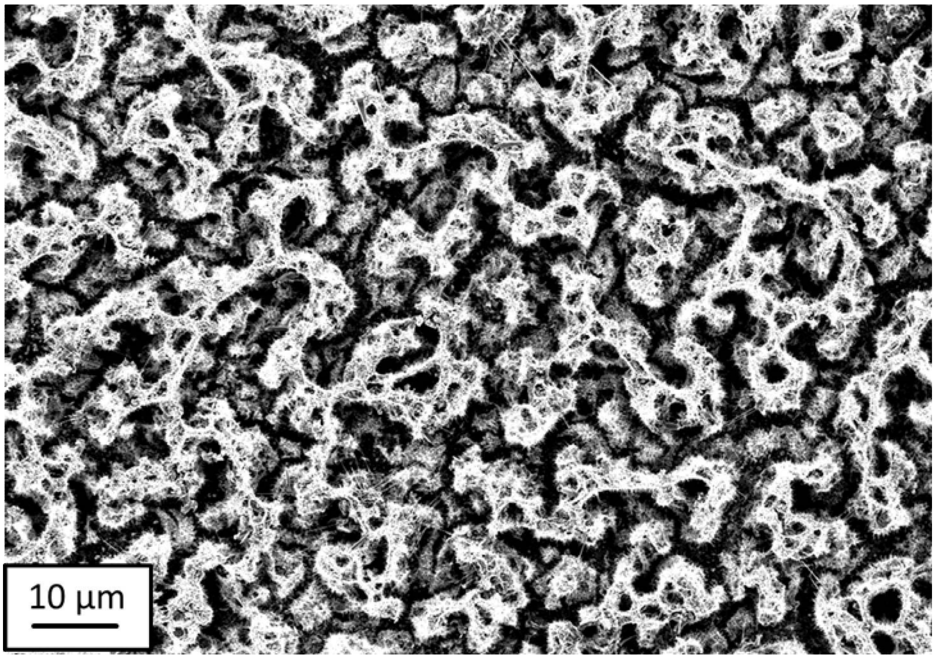

Figure S2. SEM image of SnO_2/C nanocomposite without using L-cysteine in the precursor.

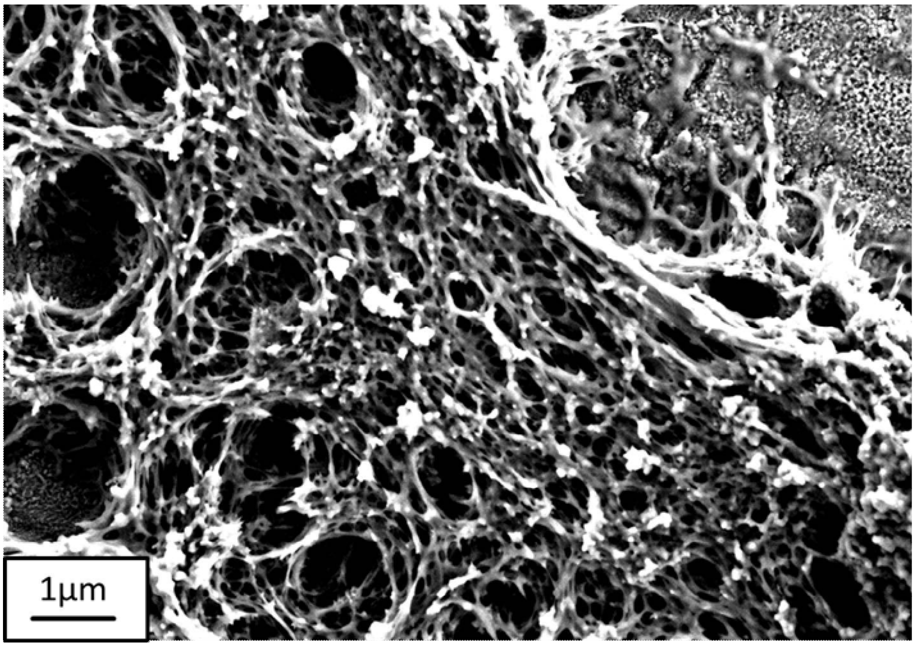

Figure S3. SEM image of carbon without using $SnCl_2$ in the precursor.

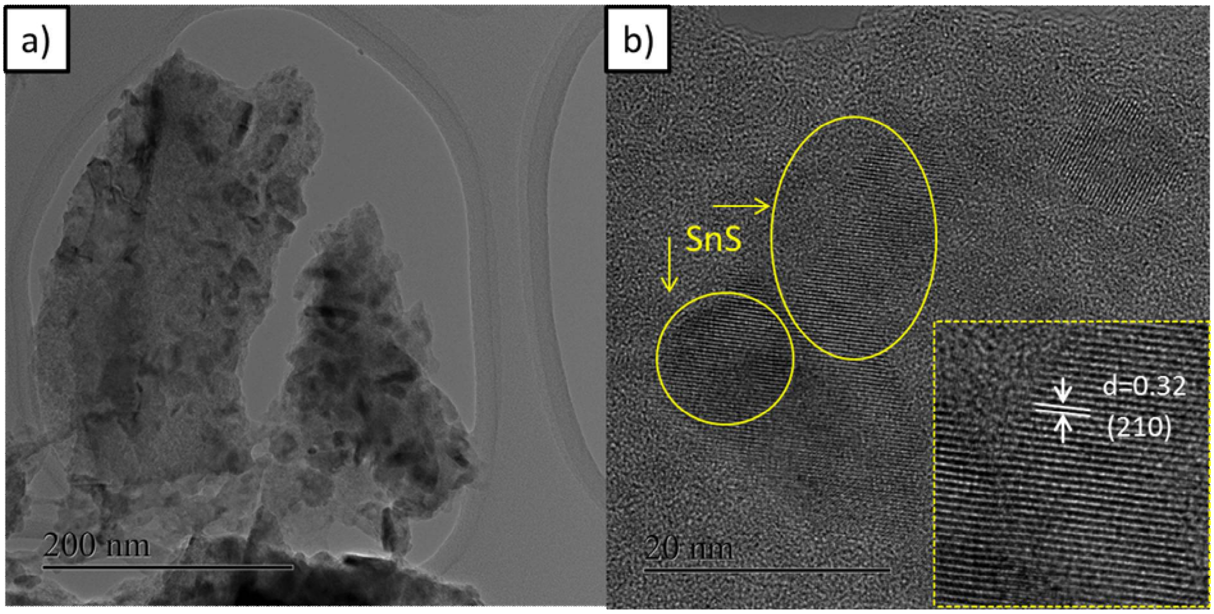

Figure S4. TEM and HRTEM images of nanoplates appearing on the surface of composite.

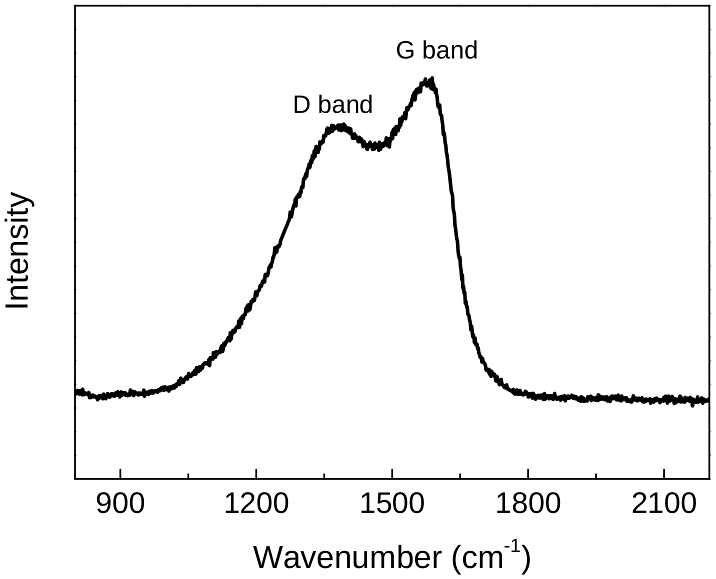

Figure S5. Raman spectra for carbon coated 3D porous interconnected SnS.

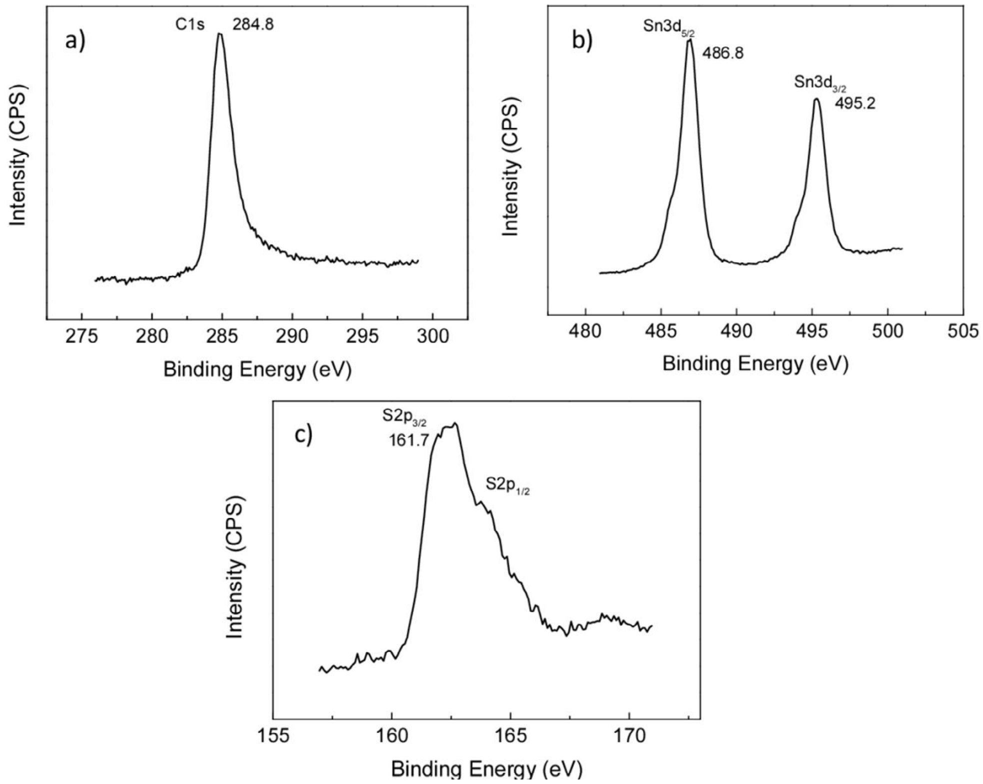

Figure S6. XPS spectra of the SnS/C nanocomposite: a) C region, b) Sn region and c) S region.

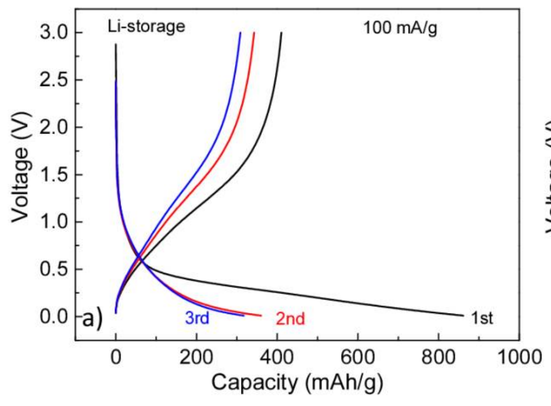

Figure S7. Charge and discharge voltage profiles for the first three cycles at current density of 100mA/g for both lithium and sodium storage for the carbon materials obtained without using $SnCl_2$ in the precursor.

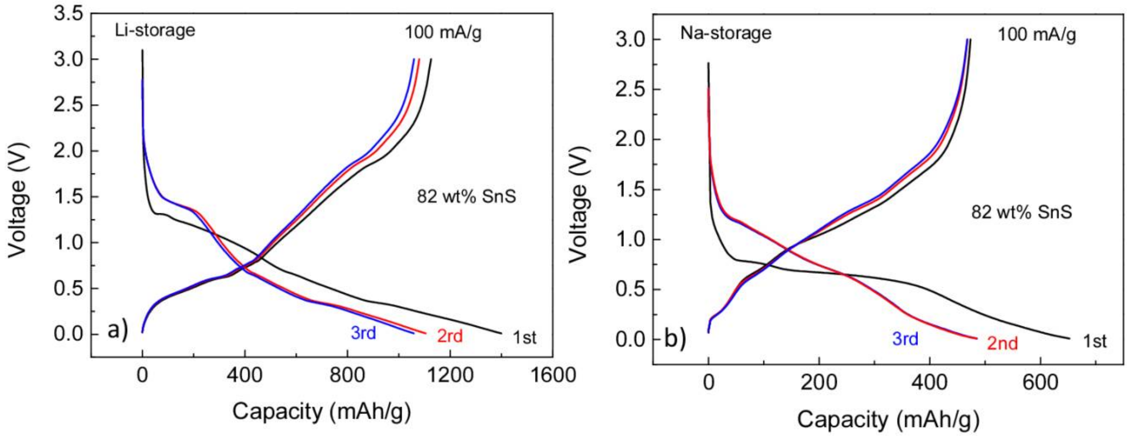

Figure S8. Charge and discharge voltage profiles for the first three cycles at current density of 100mA/g for both lithium and sodium storage for the SnS/C nanocomposite with 82wt% of SnS.

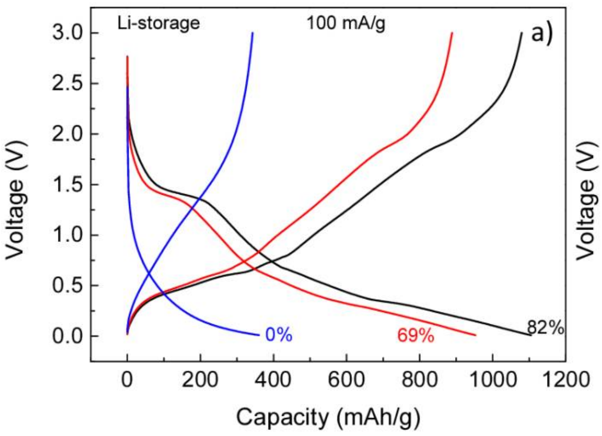

Figure S9. Comparisons of electrochemical performance of SnS/C nanocomposite with various SnS content for both lithium and sodium storage.

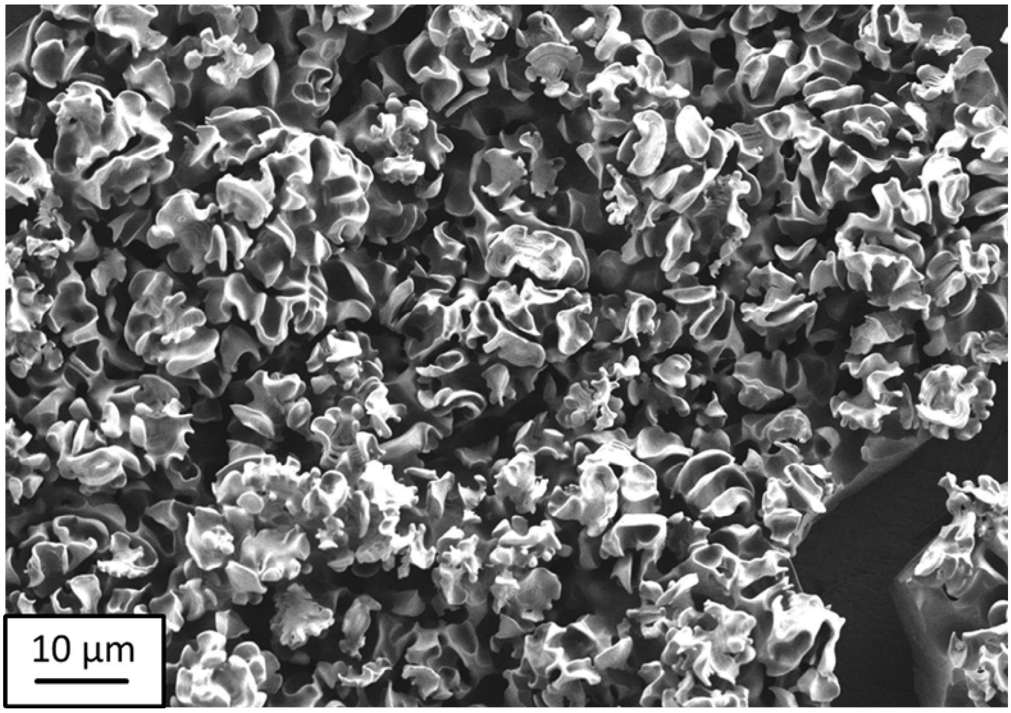

Figure S10. SEM image of 3D porous interconnected carbon coated ZnS prepared by ESD technique.
